# Long-term impact of a bonus freeze on clinical outcome: Analysis of effective and non-effective bonus freezes in cryoballoon ablation

**DOI:** 10.1371/journal.pone.0214231

**Published:** 2019-05-03

**Authors:** Buelent Koektuerk, Oezlem Koektuerk, Hikmet Yorgun, Jan-Erik Guelker, Cem Turan, Eduard Gorr, Goekmen Turan, Marc Horlitz, Paul Martin Bansmann

**Affiliations:** 1 Witten/Herdecke University, Helios Dr. Horst Schmidt Kliniken Wiesbaden, Clinic for invasive Electrophysiology and Rhythmology/ Division Cardiology II, Wiesbaden, Germany; 2 Witten/Herdecke University, Department of Medicine, Witten, Germany; 3 Department of Cardiology, Hacettepe University Faculty of Medicine, Ankara, Turkey; 4 Heart Centre Niederrhein, Department of Cardiology, Helios Clinic Krefeld, Krefeld, Germany; 5 Witten/Herdecke University, Krankenhaus Porz am Rhein, Department of Cardiology / Electrophysiology, Cologne, Germany; 6 Witten/Herdecke University, Krankenhaus Porz am Rhein, Department of Radiology, Cologne, Germany; Klinikum Region Hannover GmbH, GERMANY

## Abstract

**Purpose:**

Data on bonus freeze characteristics and their impact on complication rates and long-term clinical outcome are limited.

**Methods:**

Pulmonary vein isolation (PVI) using a 28 mm 2^nd^-generation cryoballoon (CB) was performed in 169 patients (pts). The isolation temperatures, time to isolation and minimal temperatures of the cryoapplications were documented.

**Results:**

The study included 92 pts who received one bonus freeze after PVI in group I and 77 pts who did not receive a bonus freeze in group II. After a mean follow-up time of 19.0±8.6 months in group I and 16.4±7.5 months in group II, 67 of 92 pts (72.8%) and 49 of 75 pts available to follow up (65.3%; p = 0.221) were free of atrial tachyarrhythmia, respectively. Phrenic nerve palsy occurred in 5.4% of the pts in group I (5/92 pts) and 1.3% of the pts in group II (1/77 pts; p = 0.22). Both the mean nadir temperatures of the bonus freezes and mean nadir temperatures of the isolation freezes differed significantly between the recurrent and non-recurrent pts in group I. The predilection sites of the reconduction for both groups were the inferior aspect of the inferior pulmonary veins.

**Conclusion:**

The impact of a bonus freeze on long-term clinical outcome was not significant for two reasons: 1) The necessity of a bonus freeze was low because the long-term clinical success rate without a bonus freeze was high; and 2) the majority of bonus freezes, especially at the predilection sites, such as the inferior PV, appeared to be ineffective.

## Introduction

Pulmonary vein isolation (PVI) using a 2^nd^-generation cryoballoon (CB) has been shown to be a safe and successful therapy option in patients with paroxysmal and persistent atrial fibrillation.[1–4] The second generation of CBs has been repeatedly demonstrated to produce superior long-term clinical effects compared with the first generation of CBs.[5,6] Sustained transmural and contiguous lesion formation without gaps is mandatory for the long-term success of PVI. To achieve this goal in cryoablation, a sufficiently low minimal temperature, shorter application time, slow thawing, fast cooling rate, proper CB-tissue contact, PV occlusion and repetition of the freeze-thaw cycle is recommended.[7–9] Recently, Ciconte et al. demonstrated that a single 3-minute freeze without a bonus freeze resulted in a clinical success rate of 80.4% at the 1-year follow-up,[10] and other studies without a bonus protocol showed similar favorable results.[11,12] The aim of this study was to evaluate the impact of a bonus freeze on the complication rates, long-term clinical outcome and predilection sites in cases of LA-to-PV reconnections using a second-generation 28 mm CB for PVI in patients with paroxysmal atrial fibrillation.

## Methods

### Study population

In this retrospective study patients with symptomatic paroxysmal AF were scheduled between August 2012 and September 2014 for PVI using second-generation CB. AF episodes lasting less than 7 days were defined as paroxysmal according to the latest guidelines.[7,13] The study population was separated into two groups. In group I, the pulmonary veins were electrically isolated, and an additional application (the so-called “bonus freeze”) was then applied. In group II, the final application was the isolation freeze without the bonus freeze protocol. The selection of patients according to the bonus or non–bonus protocol was a function of time; the first 92 patients had bonus freeze in the beginning of the study and the following 77 patients had non-bonus freeze protocol, respectively. The exclusion criteria included a history of severe valvular disease, persistent or longstanding persistent atrial fibrillation, decompensated heart failure, thrombus in the LA, uncontrolled thyroid dysfunction, LA diameter >50 mm, pre-procedural significant coronary artery disease (CAD), pregnancy, contraindication to anticoagulation, and attempted AF ablation. Informed consent was obtained from each patient before enrollment. The study was performed in compliance with the principles outlined in the Declaration of Helsinki and approved by the local ethics committee Ethik-Kommission der Universität Witten/Herdecke. Data cannot be made publicly available and all analyses must be approved by the principal investigator due to consent form restrictions.

### Preprocedural management

One week prior to the procedure, all of the patients underwent preprocedural diagnostic tests, such as transthoracic echocardiography (TTE), to evaluate their left ventricular ejection fraction (LVEF) and LA diameter and examine for any structural abnormalities and valvular disease. Thrombus in the LA appendage was ruled out by transesophageal echocardiography several hours prior to the procedure. In patients with anticoagulation treatment using vitamin K antagonists, the international normalized ratio (INR) was evaluated. In cases of INR levels between 2 and 3, the treatment was not terminated. Novel oral anticoagulants (NOAC), were discontinued after 24 hours, and antiarrhythmic drugs were discontinued five half-lives before the procedure.

### Index procedure with a cryoballoon

The procedures were performed under deep sedation using continuous infusion of propofol, and boluses of midazolam and fentanyl were applied when needed. A Seldinger technique was used for right femoral vein puncture. Rotational angiography was performed with an Artis C-arm system (Siemens, Erlangen, Germany) to mark the LA anatomy with pulmonary veins before accessing the LA. After placement of a 6Fr steerable decapolar and non-steerable quadripolar catheter into the coronary sinus and His region, respectively, a single transseptal puncture with a modified Brockenbrough technique (BRK-1, St Jude Medical) was performed under fluoroscopy. Left atrial and pulmonary vein angiography were performed at 40 degrees left anterior oblique and 30 degrees right anterior oblique, respectively. An activated clotting time between 300 s and 350 s was achieved with applications of heparin boluses. In all pts, a 28 mm second-generation CB catheter (Arctic Front Advance, Medtronic Inc., Minneapolis, MN, USA) was used. The PV potentials were recorded using the Achieve Mapping Catheter (Medtronic). Proper balloon occlusion was assessed by contrast injection through the catheter’s central lumen, which has been previously described in detail.(4) The duration of each freezing cycle was 240 seconds. When PV isolation failed, additional freeze was performed until complete isolation was achieved. The application resulting in PVI was called an “isolation freeze." In group I, an additional application, the so-called “bonus freeze," was performed after a PVI, whereas in group II, the bonus freeze protocol was not applied; therefore, the isolation freeze was the last application. Diaphragmatic stimulation by pacing the ipsilateral phrenic nerve with a 1,000 ms cycle and a 12mA output with a decapolar catheter placed at the superior vena cava was performed to avoid phrenic nerve palsy (PNP). Phrenic nerve capture was controlled by tactile feedback with placing the operator's hand on the patient's abdomen and intermittent fluoroscopy or diaphragmatic potentials during ECG recording as previously described in detail.

### Postprocedural management

Pericardial effusion was excluded immediately after the procedure and the day after the procedure by a TTE and. In all patients, cardiac enzyme (troponin I, CK-MB and CK) levels were determined in the serum 18 h after the procedure. After excluding a pericardial effusion an anticoagulation regime was restarted at the evening of ablation and continued for at least 3 months after the procedure. Antiarrhythmic drug therapy was also restarted for at least 3 months, and the decision for retreatment with AAD´s after 3 months was made according to the recurrence of atrial tachyarrhythmia.

### Follow-up and redo procedures

All patients were scheduled for visits in the outpatient clinics or to a referring physician at 1 month, 3 months, 6 months and 12 months after ablation or earlier if their symptoms were consistent with recurrent atrial tachyarrhythmia. A 7-day Holter recording was conducted at 3 months and 6 months, and a 24-h Holter was scheduled thereafter. Telephone interviews were performed at the end of the follow-up period before analysis. The continuation of oral anticoagulation was evaluated in the third month based on the CHA_2_DS_2_-VASc score. Recurrence was defined as any episode of AF or atrial tachyarrhythmia lasting at least 30 seconds and 3 months after the procedure was considered a blanking period. Recurrence occurring in the blanking period was classified as early, whereas recurrence after the blanking period was defined as late recurrence. The primary endpoint, respectively outcome, was defined as the absence of late recurrence of atrial fibrillation or atrial tachycardia lasting more than 30 seconds. Secondary endpoints were defined as procedure-related complications, procedure duration and fluoroscopy time. The repeat procedure was performed using either a 28 mm 2^nd^-generation CB or a Carto or EnSite NavX three-dimensional mapping system (Biosense Webster, Inc., Diamond Bar, CA, USA; St. Jude Medical, Inc., St. Paul, MN, USA, respectively). Electrical activity was evaluated at each PV using an Achieve Mapping Catheter (Medtronic) or a spiral catheter Lasso, Biosense-Webster and Spiral, St. Jude Medical) when the three-dimensional mapping system was employed. For radiofrequency ablation, a 3.5 mm irrigated-tip catheter was used. If all of the PVs were isolated, the fractionated ostial potentials were ablated.

### Statistical analysis

Normally or asymptotically normally distributed continuous parameters are presented as the mean±standard deviation and categorical data are presented as frequencies and percentages. Comparisons among the baseline characteristics were performed by an independent Student’s t test, Fisher’s exact test or a chi-squared test where appropriate. A Kaplan-Meier analysis was performed to describe AF-free survival using a log-rank-test to show differences between the two groups. Statistical analyses were performed using SPSS statistical software (version 21.0; SPSS Inc., Chicago, IL, USA). A two-tailed p-value <0.05 was considered statistically significant.

## Results

### Patient characteristics

**Tables 1 and 2**show the patients’ clinical and procedural characteristics. In both groups, a significant difference was observed in gender (group I: male n = 79 [85.9%]; group II: male n = 49 [63.6%]; p = 0.001) and age (group I: 64±10 years; group II: 61±10 years; p = 0.047), although significant differences were not observed among the cardiovascular risk factors in terms of hyperlipidemia, hypertension or diabetes mellitus. In group 1, a history of coronary artery disease was observed in 19 patients (20.7%), whereas in group 2, a history of coronary artery disease was observed in 10 patients (13.0%). The mean ejection fraction and the LA diameter for groups I and II were 61.5±6.8 and 62.6±4.6, respectively (p> 0.05), and 31.1±9.4 mm and 32.5±8.5 mm (p>0.05), respectively.

**Table 1:** Basal clinical and demographic characteristics of both study populations.

	Group I (n = 92)	Group II (n = 77)	p-value
Age (years)	64± 10	61±10	**0.047**
Male (%)	79 (85.9%)	49 (63.6%)	**0.001**
EF	61.5±6.8	62.6 ±4.6	0.594
LA (mm)	31.1±9.4	32.5 ±8.5	0.595
Hypertension (%)	53 (57.6%)	38 (52.8%)	0.635
Hyperlipidemia (%)	23 (25.0%)	13 (18.1%)	0.344
Diabetes mellitus (%)	5 (5.4%)	3 (4.2%)	0.708
CAD (%)	19 (20.7%)	10 (13.9%)	0.306
**Antiarrhythmic treatment at time of follow-up**			
Amiodarone	2 (2.2%)	0 (0.0%)	0.501
Flecainid	4 (4.3%)	4 (5.2%)	0.999
Propafenone	0 (0.0%)	0 (0.0%)	1.000
Dronedarone	1 (1.1%)	0 (0.0%)	0.999

AF: atrial fibrillation; CAD: coronary artery disease; EF: ejection fraction; LA: left atrium

**Table 2:** Procedural characteristics of both study groups.

	Group I (n = 92)	Group 2 (n = 77)	p-value
Mean number of LSPV appl.	1.4±0.8	1.5±0.7	0.319
Mean number of LIPV appl.	1.1±0.5	1.2±0.5	0.067
Mean number of RSPV appl.	1.3±0.6	1.5±1.0	0.143
Mean number of RIPV appl.	1.3±0.6	1.2±0.5	0.626
Procedure time (minutes)	102.0±23.8	71.8±22.8	<0.001
Fluoroscopy duration (minutes)	20.2±7.9	13.1±5.8	<0.001

appl.: application LIPV: left inferior pulmonary vein; LSPV: left superior pulmonary vein; RIPV: right inferior pulmonary vein; RSPV: right superior pulmonary vein

### Procedure parameters at the index CB procedure

The acute endpoint of complete pulmonary vein isolation was achieved in all 169 pts. Left common ostia (LCPV) was present in four patients in each of the two groups. Every branch of these common ostia was isolated separately. There were no significant differences in the average number of cryoenergy applications per pulmonary vein excluding the bonus freeze as shown in **Table 2**. The fluoroscopy time and procedure time differed significantly for groups I and II (20.2±7.9 and 13.1±5.8 minutes [p<0.001] and 102.0±23.8 and 71.8±22.8 minutes [p<0.001], respectively).

### Cardiac enzymes

The troponin I-, CK-MB- and CK-levels in the serum increased 24 h after the procedure but were not significantly different in the two groups. In the subgroup of group II, in which only 4 applications for the whole procedure (only a single application for each pulmonary vein) were applied, the cardiac enzymes were increased compared to group I but not significantly different (**Table 3**).

**Table 3:** Cardiac enzymes 18 h after intervention.

	Group I	Group II	Subgroup II (4 total appl.)	p-value
Troponin I (ng/ml)	7.6±3.6	7.0 ±4.7	6.4±2.8	n.s.
CK-MB (U/l)	47.0±23.4	47.6±25.6	43.7±11.9	n.s.
CK (U/l)	303.2±172.2	317.1 ±171.5	334.9±226.7	n.s.

CK = creatine kinase; CK-MB = creatine kinase-MB

### Complications

Adverse events or complications were observed as right phrenic nerve palsy and hematoma. PNP occurred in 5.4% of the pts in group I (5/92 pts) and 1.3% of the pts in group II (1/77 pts) (p = 0.22). All PNP in group I occurred during the bonus freeze. Chest fluoroscopy performed 3–8 months after the index procedure showed a complete resolution of the PNP. In 5 patients, vascular access problems in terms of hematoma in the right groin occurred, although surgical intervention was not necessary. Pericardial effusion, pericardial tamponade or cerebrovascular events were not observed during or after the procedures.

### Follow-up and repeat procedures

**Fig 1:**
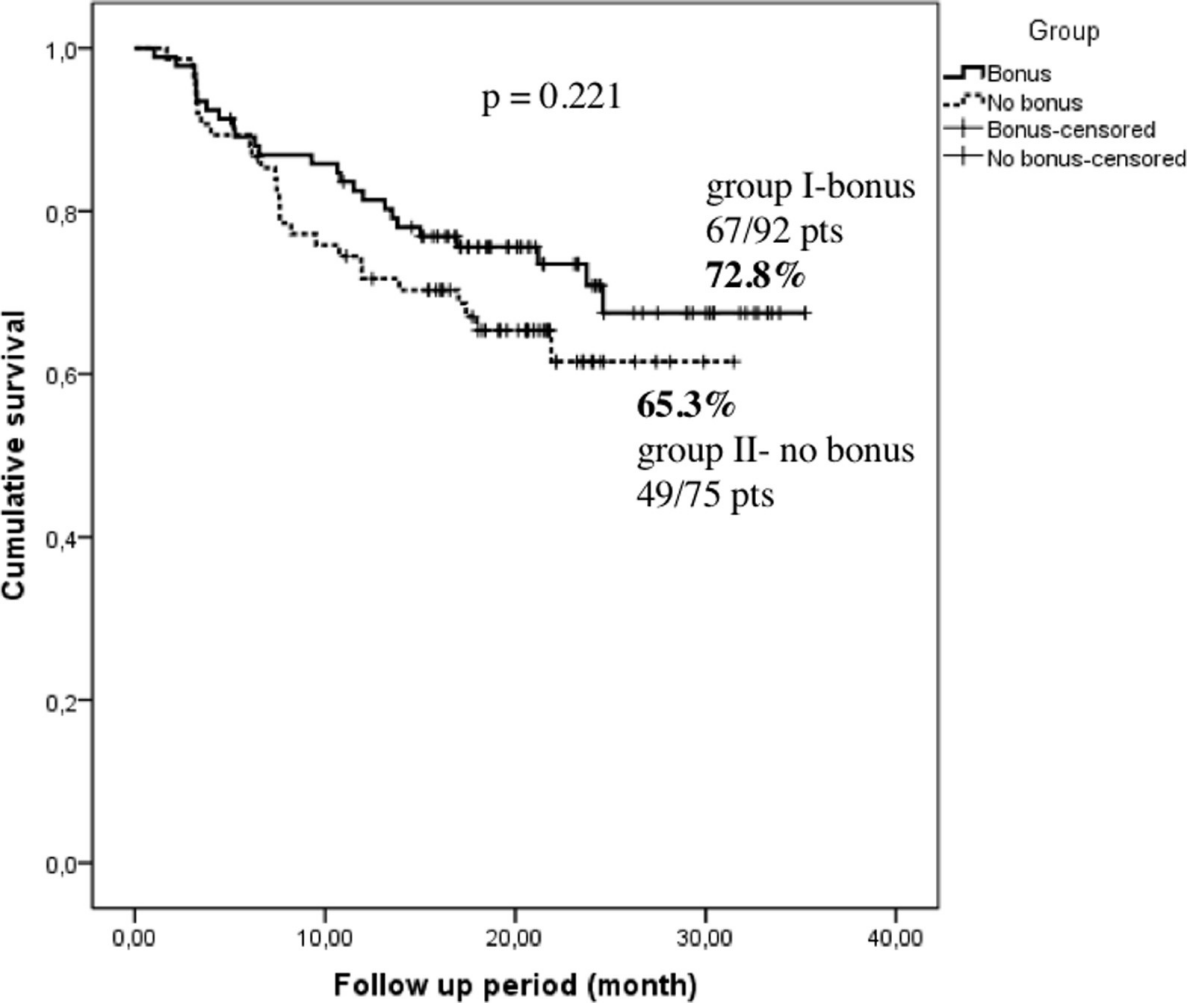
Freedom from atrial fibrillation for a mean follow-up time of 19.0±8.6 months in group I and 16.4±7.5 months in group II following second-generation cryoballoon ablation when a 3 month blanking period was considered.

After a mean follow-up time of 19.0±8.6 months in group I and 16.4±7.5 months in group II, 67 of 92 pts (72.8%) and 49 of 75 pts (65.3%) were free of atrial tachyarrhythmia, respectively. The atrial tachyarrhythmia-free survival was not statistically significant (p = 0.221), although group I tended to have a better clinical outcome as shown in the Kaplan-Meier curve in **Fig 1**. At the end of the follow-up time, 6 of 92 (6.5%) pts in group I and 4 of 75 (5.3%) pts in group II without recurrence of AF were on antiarrhythmic therapy, whereas one patient in group I and no patients in group II with recurrent atrial tachyarrhythmia were on antiarrhythmic drugs **(Table 1)**. Atrial tachycardia after the index procedure was not documented.

**Fig 2:**
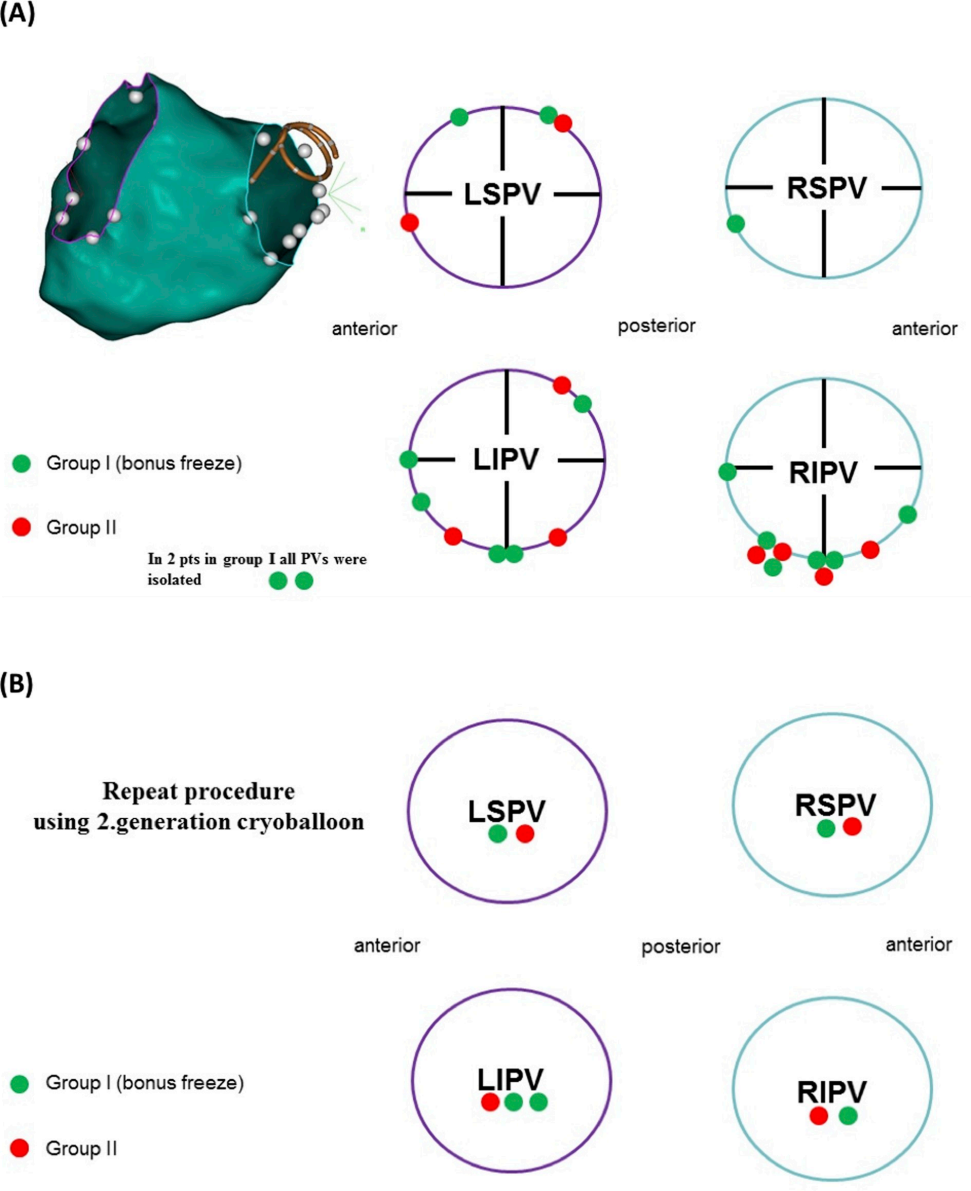
Reconduction sites (gaps) for each pulmonary vein: Green and red dots depict the localization of the reconnections in group I and group II, respectively. (A) Repeat procedures performed with a 3D mapping system and ablated with irrigated RF energy (in 2 pts from group II, all PVs still were isolated; and (B) Repeat procedures performed with the 28 mm second-generation cryoballoon. LIPV: left inferior pulmonary vein; LSPV: left superior pulmonary vein; RIPV: right inferior pulmonary vein; RSPV: right superior pulmonary vein.

Of the 25 pts in group I and 19 pts in group II with recurrent atrial fibrillation, 14 and 7 pts underwent a repeat procedure (83 PVs), respectively. In 6 pts, the repeat procedure was performed using a 28-mm second cryoballoon, and in 15 pts, the procedure was performed using a three-dimensional mapping system using RF energy (**Fig 2**). In groups I and II, 19 of 56 PVs (34%) and 13 of 27 PVs (48%) were reconnected, respectively. In both groups, the predilected sites of reconnection were at the inferior segment of the inferior pulmonary veins (72%). In 2 pts of group I undergoing a repeat procedure, all of the pulmonary veins were still electrically isolated (**Fig 2A**). Reconduction of more than 1 PV per patient could be demonstrated in 6 of 14 pts (42.9%) in group I, whereas reconduction of more than 1 PV was observed in 5 of the 7 pts (71.4%; p = 0.316) in group II.

### Temperature monitoring and isolation characteristics

**Fig 3:**
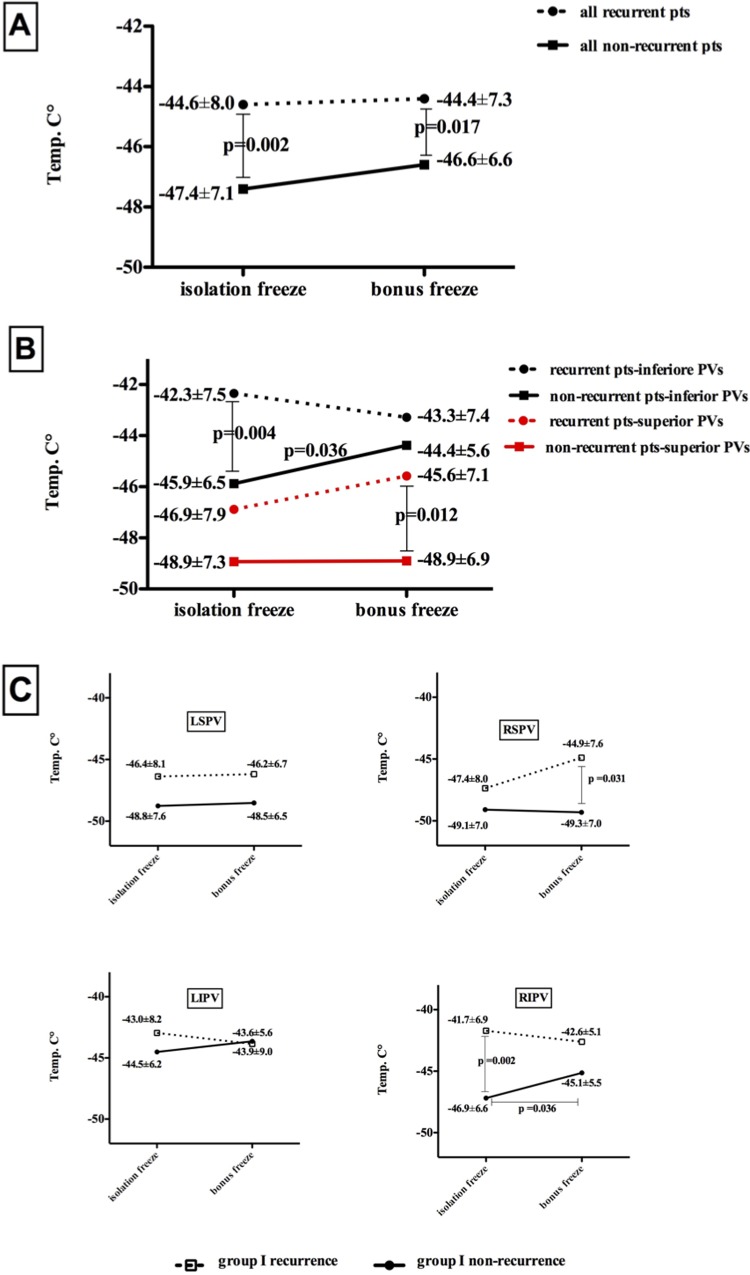
Mean minimal temperature of the "isolation" freezes and consequent bonus freezes in group I: Separated by recurrent and non-recurrent patients of group I. (A) All pts in group I; (B) pts grouped according to inferior (LIPVs and RIPVs) and superior (LSPVs and RSPVs) pulmonary veins; (C) pts separated by single PVs (LSPV, RSPV, LIPV, and RIPV). LIPV: left inferior pulmonary vein; LSPV: left superior pulmonary vein; RIPV: right inferior pulmonary vein; RSPV: right superior pulmonary vein.

In group I, the mean minimal temperature of the isolation freeze in all recurrent patients was significantly higher (warmer) than that in the non-recurrent pts (**Fig 3A**; recurrent pts [-44.6°C±8.0°C] vs non-recurrent pts [-47.4°C±7.1°C]; p = 0.002). Significant differences were also observed between the nadir temperatures of the bonus freezes in the recurrent and non-recurrent pts (**Fig 3A**; recurrent pts [-44.4°C±7.3°C] vs non-recurrent pts [-46.6°C±6.6°C]; p = 0.017).

Recurrences in the inferior PVs (LIPVs and RIPVs) in group I were associated with significantly higher (warmer) nadir temperatures of the isolation freeze (**Fig 3B**; recurrent pts [-42.3°C±7.5°C] vs non-recurrent pts [-45.9°C±6.5°C]; p = 0.004). The mean bonus freeze temperature in the inferior PVs of the non-recurrent pts was significantly higher (warmer) relative to the isolation freeze in these pts (**Fig 3B**; -45.9°C±6.5°C vs -44.4°C±5.6°C; p = 0.036). The nadir temperatures of the bonus freezes of the superior PVs differed significantly between the recurrent and non-recurrent pts (**Fig 3B;** -45.6°C±7.1°C vs -48.9°C±6.9°C; p = 0.012).

In the pulmonary vein-based analysis, statistical significance was reached for the minimal isolation freeze temperature of the RIPVs (**Fig 3C**; recurrent pts [-41.7°C±6.9°C] vs non-recurrent pts [-46.9°C±6.6°C]; p = 0.002), and this result was also observed in the repeat procedures, with the reconnected RIPVs in both groups associated with significantly higher minimal temperatures in the index CB procedure compared the still isolated RIPVs (mean minimal temperature of the reconnected RIPVs at -41.2°C±3.4°C; the mean minimal temperature of still isolated RIPVs at -46.4°C±7.3°C; p<0.001). Significant differences were not observed between the isolation times and temperatures for the remaining PVs in group I and the PVs in group II **(Table 4**). The mean minimal temperature of the bonus freeze was not significantly lower (colder) than the previous isolation freeze in any of the PVs.

**Table 4:** Cooling and isolation characteristics at the index procedure performed with a second-generation cryoballoon, and the results are separated by group I and group II and divided by recurrence and non-recurrence patients.

	Group	Recurrence (Mean)		p-value
		Yes	No	
LSPV isolation temperature	I	-33.9±10.3	-36.6±6.3	0.350
	II	-36.4±10.0	-39.2±5.9	0.251
LSPV time to isolation	I	51.6±30.2	50.9±23.9	0.943
	II	58.7±28.1	66.1±34.6	0.526
LSPV minimum temperature	I	-46.4±8.1	-48.8±7.6	0.222
	II	-45.6±8.6	-47.9±6.2	0.237
LIPV isolation temperature	I	-34.0±12.4	-33.1±8.0	0.810
	II	-30.0±8.2	-31.1±10.1	0.717
LIPV time to isolation	I	49.1±26.2	58.9±35.3	0.495
	II	53.5±43.2	51.0±46.2	0.858
LIPV minimum temperature	I	-43.0±8.2	-44.5±6.2	0.357
	II	-42.1±5.3	-43.4±6.4	0.479
RSPV isolation temperature	I	-45.0±20.7	-35.6±9.7	0.123
	II	-34.8±12.9	-38.2±8.0	0.414
RSPV time to isolation	I	59.8±56.3	55.0±40.6	0.824
	II	53.4±44.2	56.5±39.1	0.833
RSPV minimum temperature	I	-47.4±8.0	-49.1±7.0	0.339
	II	-49.8±7.1	-48.1±17.4	0.405
RIPV isolation temperature	I	-31.2±14.3	-36.1±8.8	0.306
	II	-34.7±5.9	-38.2±6.2	0.106
RIPV time to isolation	I	66.2±45.7	66.8±44.5	0.979
	II	69.6±42.8	72.9±41.7	0.820
RIPV minimum temperature	**I**	**-41.7**±**6.9**	**-47.2**±**6.6**	**0.002**
	II	-46.9±8.6	-46.1±7.2	0.710

LIPV: left inferior pulmonary vein; LSPV: left superior pulmonary vein; RIPV: right inferior pulmonary vein; RSPV: right superior pulmonary vein

## Discussion

### Main findings

The major findings of our study were as follows: (1) The bonus freeze did not significantly improve the long-term clinical outcome; however, there was a tendency towards a better clinical outcome and reduction in the total number of PV reconductions per patient in group I. (2) The procedure times and fluoroscopy times in group II were significantly shorter. (3) The mean nadir temperatures of the bonus freezes and the mean nadir temperatures of the isolation freezes differed significantly between the recurrent and non-recurrent pts in group I. (4) The bonus freeze did not change the predilection sites for PV reconduction, and for both groups, the inferior segment of the inferior pulmonary veins was the preferred localization for gaps (5) Finally, the bonus freeze at the inferior PVs was, in most cases, ineffective, as revealed by the minimal temperature.

### Previous studies

Previous studies analyzing first-generation CBs showed that after PVI, a second bonus freeze increased the complication rates without improving clinical success compared with only one bonus freeze.[14] To our knowledge, a protocol without a bonus freeze has never been investigated and evaluated for first-generation CBs. In the SUPIR study, Reddy et al. showed that the durability rate of PVI using a 2^nd^-generation CB was 91%; however, a bonus freeze protocol was applied with a duration of 240 ms per application, and it was not compared with a no-bonus protocol.[15] Recently, several studies have shown a long-term success rate of 80%–82% after a follow-up time of one year after performing a protocol without a bonus freeze using a 2^nd^-generation CB.[10–12] However, the clinical success of PVI in patients with paroxysmal atrial fibrillation is not always correlated with durable electrical isolation.[16] Studies have shown that asymptomatic patients after PVI can have gaps and electrical reconduction of the pulmonary veins.[15,17] In our study, 2 pts of group I with recurrent paroxysmal atrial fibrillation and a repeat procedure presented PVs that were all still isolated. However, in re-mapping studies of symptomatic pts, a high rate of reconnection of one or more PVs and durable electrical isolation of all PVs were generally associated with a better clinical outcome.[15,17] Using a 2^nd^-generation cryoballoon with an optimized catheter design and cooling properties, the incidence of persistent isolated PVs was high.[9,18] In the majority of PVs, the application of a bonus freeze to increase the long-term outcome did not appear to be necessary. Therefore, the benefits of a bonus freeze might only be observed a small number of PVs after electrical isolation. A limited number of studies have evaluated second re-mapping procedures to determine the impact of a bonus freeze after PVI using a second generation CB relative to the benefits of a no-bonus freeze procedure. In two recently published studies, statistically significant differences were not observed between the bonus and no-bonus freeze protocols regarding the durability of the isolated PVs.[9,18] In one study, the number of pts was too small for a definitive conclusion,[18] whereas in the second study, statistically significant differences were not observed between the bonus freeze protocol and no-bonus freeze protocol in terms of durable isolated PVs; thus, an additional freeze was suggested to be beneficial if the isolation freeze was not associated with acute fast PVI or achievement of -40°C within 60 seconds.[9] These results are similar to the results of our study and might be related to the ineffective nature of the majority of bonus freezes as isolation freezes in reconnected PVs and the lack of additional contributions to the durability of the PVI.

### Predilection sites of PV reconduction

In this study, the preferred sites for both groups were the inferior aspects of the inferior PVs, especially the right inferior pulmonary vein. The minimal temperature of the RIPV for recurrent pts in group I and for all pts with reconducted RIPVs was statistically higher (warmer) compared with that of the non-recurrent pts in group I and the still-isolated RIPVs. Experimental studies have previously shown that the minimal temperature is a crucial determinant for durable PVs.[19] Whether a possible incomplete CB contact at the tissue and/or improper PV occlusion could play a role in achieving higher temperatures and manifesting these predilection sites should be investigated in further studies. Gosh et al. previously reported that in addition to the balloon warming time, vein occlusion is an independent predictor for late pulmonary vein reconnection when using a first-generation CB, and they suggested improved techniques for optimizing balloon/tissue to increase the rate of long-term durable PVIs.[20] Moreover, whether a pull-down maneuver plays a role in the manifestation of gaps in inferior PVs remains to be investigated.

### Bonus freeze characterization

Cryoablation lesion formation can be divided into three components: the freeze/thaw phase, the hemorrhagic-inflammatory phase and the replacement fibrosis phase.[21] Cell damage is mediated by a number of determinants, such as the minimal temperature, application time, fast freezing rate, slow warming rate, area and duration of contact tissue as well as thermal conductivity.[7,8] If one of these factors is insufficient, the cells can reach a sublethal status if the other determinants do not provide compensation. If permanent injury is not achieved, then the cells can recover.[22,23] The sublethal status of the cells and presentation of edema are important factors leading to an acute PVI with later reconduction. In such cases, a bonus freeze can be beneficial because such procedures have been reported to increase the lesion boundaries and necrosis.[8,24] Repeated freeze/thaw cycles are known to increase cell destruction.[25] However, in our study, the cardiac enzyme levels that indicate cardiac damage did not differ significantly between the groups. Even in the subgroup of group II, in which only 4 applications were performed for the entire procedure, the volume of cell necrosis or apoptosis was not significantly different, and similar troponin I or CK-MB-level elevations were observed across the groups. This result indicated that the bonus freeze was performed in the same tissue area, did not require a large change in the placement of the CB, and presented similar CB tissue contact and PV occlusion. In such cases, a bonus application did not significantly change the lesion boundaries.

Our study demonstrated that for pts without recurrence at predilection sites (inferior PVs), significantly lower (colder) minimal isolation freeze temperatures were observed compared with that in the recurrent pts. A bonus freeze at the inferior PVs was ineffective in most cases as revealed by the minimal temperature for the both recurrent and non-recurrent pts. In the non-recurrent pts, the nadir bonus freeze temperatures of the inferior PVs were significantly higher (warmer) compared with that of the isolation freezes, which could be interpreted as ineffective bonus freezes. However, in the recurrent pts, the bonus freeze of the inferior PVs did not contribute to a better long-term outcome, which was similar to the results of an insufficient isolation freeze as revealed by the lack of significantly different mean minimal temperatures. Therefore, for both the recurrent and non-recurrent pts, bonus freezes in the inferior PVs can be considered ineffective. The minimal temperatures of the isolation freezes at the superior PVs were not significantly different; however, the bonus freeze temperatures between the recurrent and non-recurrent pts differed significantly. At the superior PVs, an additional beneficial contribution of the bonus freezes was possible, although because of the small number of repeated procedures, a definitive conclusion could not be made.

### Limitations

This study was an observational investigation and not a randomized study. In addition to evaluating the clinical success and recurrence rates, the analysis was also dependent on the follow-up methods. We used 7-day Holter ECGs, which are not as sensitive as ICMs but are more sensitive than 24-h Holter ECG recordings. Nevertheless, randomized prospective studies with continuous intracardiac monitoring and second mapping procedures should be performed to investigate the correlation between a bonus freeze and the durable PVI and between a bonus freeze and the lack of atrial tachyarrhythmia. Asymptomatic episodes of atrial tachyarrhythmias are more accurately documented by ICM.

## Conclusions

The impact of a bonus freeze on the long-term clinical outcome was not significant for two reasons: 1) The necessity for a bonus freeze is low because the long-term clinical success rate without a bonus freeze is high; and 2) The majority of the bonus freezes, especially at the predilection sites, such as the inferior PV, appear to be ineffective. Further studies should investigate predictors for effective bonus freezes in terms of durable PVIs after acute-successful but long-term insufficient isolation freezes.
